# Exposure to GABA_A_ Receptor Antagonist Picrotoxin in Pregnant Mice Causes Autism-Like Behaviors and Aberrant Gene Expression in Offspring

**DOI:** 10.3389/fpsyt.2022.821354

**Published:** 2022-02-03

**Authors:** Hiroko Kotajima-Murakami, Hideo Hagihara, Atsushi Sato, Yoko Hagino, Miho Tanaka, Yoshihisa Katoh, Yasumasa Nishito, Yukio Takamatsu, Shigeo Uchino, Tsuyoshi Miyakawa, Kazutaka Ikeda

**Affiliations:** ^1^Addictive Substance Project, Tokyo Metropolitan Institute of Medical Science, Setagaya-Ku, Japan; ^2^Department of Biosciences, School of Science and Engineering, Teikyo University, Utsunomiya-Shi, Japan; ^3^Division of Systems Medical Science, Institute for Comprehensive Medical Science, Fujita Health University, Toyoake-Shi, Japan; ^4^Department of Pediatrics, Graduate School of Medicine, The University of Tokyo, Bunkyo-Ku, Japan; ^5^Department of Psychiatry, The University of Tokyo Hospital, Bunkyo-Ku, Japan; ^6^Department of Obstetrics and Gynecology, Graduate School of Medicine, The University of Tokyo, Bunkyo-Ku, Japan; ^7^Center for Basic Technology Research, Tokyo Metropolitan Institute of Medical Science, Setagaya-Ku, Japan

**Keywords:** autism spectrum disorder, picrotoxin, GABA_A_ receptor, social interaction, odorant binding, gene expression, microarray, WGCNA

## Abstract

Autism spectrum disorder (ASD) is a neurodevelopmental disorder that is characterized by impairments in social interaction and restricted/repetitive behaviors. The neurotransmitter γ*-*aminobutyric acid (GABA) through GABA_A_ receptor signaling in the immature brain plays a key role in the development of neuronal circuits. Excitatory/inhibitory imbalance in the mature brain has been investigated as a pathophysiological mechanism of ASD. However, whether and how disturbances of GABA signaling in embryos that are caused by GABA_A_ receptor inhibitors cause ASD-like pathophysiology are poorly understood. The present study examined whether exposure to the GABA_A_ receptor antagonist picrotoxin causes ASD-like pathophysiology in offspring by conducting behavioral tests from the juvenile period to adulthood and performing gene expression analyses in mature mouse brains. Here, we found that male mice that were prenatally exposed to picrotoxin exhibited a reduction of active interaction time in the social interaction test in both adolescence and adulthood. The gene expression analyses showed that picrotoxin-exposed male mice exhibited a significant increase in the gene expression of odorant receptors. Weighted gene co-expression network analysis showed a strong correlation between social interaction and enrichment of the “odorant binding” pathway gene module. Our findings suggest that exposure to a GABA_A_ receptor inhibitor during the embryonic period induces ASD-like behavior, and impairments in odorant function may contribute to social deficits in offspring.

## Introduction

Autism spectrum disorder (ASD) is categorized as a neurodevelopmental disorder in the *Diagnostic and Statistical Manual of Mental Disorders*, 5th edition (1). Although ASD has several peripheral symptoms (e.g., aberrant sensitization and clumsiness of movement), characteristics of ASD are divided into two main categories: impairments in social interaction and communication and restricted and repetitive patterns of behaviors and interests (1). Symptoms of ASD are usually diagnosed during early childhood and remain during an individual's life. The ratio of the prevalence of ASD in males and females is ~4:1 (2). Genetic and environmental causes of ASD have been investigated (3, 4), but the pathophysiology of ASD has not yet been thoroughly defined.

γ-Aminobutyric acid (GABA) is an inhibitory neurotransmitter in the mature brain that hyperpolarizes a membrane through the influx of chloride ions *via* GABA_A_ receptor channels (5). GABA_A_ receptor activation induces depolarizing membrane responses in the immature central nervous system (CNS), and GABA is important in the development of neuronal circuits, neurogenesis, and synapse formation (5, 6). Secreted GABA increased cell proliferation in the ventricular zone through GABA_A_ receptor activation in mouse fetuses (7). Spontaneous Ca^2+^ oscillations, which are required for normal neuronal migration, are blocked or their frequency is reduced by the GABA_A_ receptor blocker bicuculine in the cerebral cortex in newborn rats (8). Neuroblast migration in the hippocampus is impaired by treatment with antagonists of GABA_A_ receptors and *N*-methyl-D-aspartate (NMDA) receptors, and GABA_A_ receptor antagonism is more efficient than NMDA receptor antagonism in reducing cell migration (9). Previous studies showed that perinatal and postnatal GABA_A_ receptor antagonist treatment led to aberrant behaviors in males. Bicuculine treatment during the neonatal period causes aberrant anxiety-like behavior in mature male mice and rats but not in females (10, 11). Male rats that were exposed to picrotoxin as embryos exhibited aberrant heterotypical sexual behaviors compared with control rats (12–14). These findings suggest that GABA signaling through GABA_A_ receptors plays a key role in development of the immature CNS, and the inhibition of GABA signaling during developmental periods causes abnormal behaviors in male offspring.

Tochitani et al. recently reported that prenatal treatment with GABA_A_ receptor agonists or antagonists altered social behaviors and locomotor activity in male offspring (15). They also reported that picrotoxin treatment from embryonic day 10–12 caused a rapid loss of interest in a familiar mouse, decreased locomotor activity, and decreased rearing (15). This study showed that disturbances of GABA_A_ receptor signaling by picrotoxin administration during the embryonic period caused pathophysiological neurodevelopmental abnormalities, including ASD-like symptoms (15). However, unclear are whether and how picrotoxin affects body maturation and behaviors from adolescence to adulthood and gene expression in the mouse brain and whether there are correlations between such behavioral alterations and gene expression.

The present study investigated the effects of prenatal exposure to the GABA_A_ receptor antagonist picrotoxin on body maturation and performance in several behavioral tests, including motor function, social interaction, pain responsiveness, self-grooming, and anxiety-like behavior. We then performed a comprehensive gene expression analysis using microarrays in the whole brain to explore the effects of picrotoxin on ASD-like pathophysiology. We also compared gene expression in the whole brain between picrotoxin-exposed mice and VPA-exposed mice (i.e., an established animal model of ASD). We analyzed data using BaseSpace and weighted gene co-expression network analysis (WGCNA). Here, we present evidence that offspring that are exposed to picrotoxin during the embryonic period exhibit impairments in social interaction in both adolescence and adulthood and that performance in the social interaction is strongly correlated with the odorant pathway in the WGCNA. Our results support the hypothesis that disturbances of GABA_A_ receptor signaling during the embryonic period contributes to the pathophysiology of ASD.

## Materials and Methods

### Mice and Picrotoxin Administration

Pregnant C57BL/6J mice were purchased from CLEA (Tokyo, Japan) on gestation day 6 and housed individually. All of the mice were housed on a 12/12 h light/dark cycle (lights on 8:00 a.m. to 8:00 p.m.) and had *ad libitum* access to food and water. Temperature was maintained at 23.0 ± 1.0°C. Picrotoxin was dissolved in saline. Pregnant female mice received a single intraperitoneal injection of 5 mg/kg picrotoxin (Sigma-Aldrich, St. Louis, MO, USA) on gestation day 12.5. The dose of picrotoxin was based on a previous study that reported that the 5.0 mg/kg dose twice daily did not cause malformation or infant death in offspring (16). We also tested 2.5 and 5.0 mg/kg doses in pregnant female mice and observed impairments in social interaction in both adolescence and adulthood only in offspring that were exposed to the 5.0 mg/kg dose (Supplementary Figure 1). Thus, in the present study, we administered 5.0 mg/kg picrotoxin in pregnant mice. Picrotoxin was injected only once on embryonic day 12.5 to avoid possible negative effects of repeated administration in pregnant mice and to avoid the possibility of causing a cleft palate in offspring (17). We chose embryonic day 12.5 for administration to compare gene expression between picrotoxin-exposed mice and mice that were prenatally exposed to VPA on embryonic day 12.5 (i.e., an established animal model of ASD) (18). Control pregnant mice were injected with saline. The volume of injection was 10 ml/kg. All pregnant mice were returned to their home cages immediately after the injection, and their conditions were observed carefully. Eleven pregnant mice were used in this experiment. Five pregnant mice were used as the control group. Six pregnant mice were used as the picrotoxin group. Five minutes after the picrotoxin injection, the movements of pregnant mice were slow, and they mostly lied down on the floor. After ~60 min, the state of pregnant mice became normal (i.e., washing their faces and walking in the cage as usual). On average, 6–10 pups were obtained from picrotoxin- and saline-treated pregnant female mice. As in our previous study (18), the pups were culled to eight animals per litter on postnatal day 4 (P4). For saline-exposed pups, for litters with <8 pups, pups were transferred from litters that had more than nine on P4. We did not observe postnatal mortality, malformation, or stunted pups. The day of birth was defined as day 0. The pups were weaned on P25, and mice of either sex were housed separately. Three to five offspring were housed in one home cage. All of the animal experiments were performed in accordance with the Guidelines for the Care of Laboratory Animals of the Tokyo Metropolitan Institute of Medical Science, and the housing conditions were approved by the Institutional Animal Care and Use Committee (approval no. 12-43).

### Postnatal Body Maturation and Behavioral Analyses

The body weights and eye opening of the mice were monitored to assess postnatal body maturation. Body weight was recorded on P7, P9, P11, P14, P21, and P25. Eye opening was observed once daily from P12 to P18. The eye-opening score was the following: 0 = both eyes closed, 1 = one eye open, and 2 = both eyes open. All of the behavioral tests were performed from 9:00 a.m. to 6:00 p.m. The mice were given 60 min to habituate to the behavioral test room before the start of each test. Motor function during from P7 to P25 was assessed by the negative-geotaxis, righting reflex, cliff avoidance, and hanging wire tests. The social interaction test was conducted in both adolescence (5–6 weeks of age) and adulthood (10–11 weeks of age). The mice underwent the hot plate test (6–7 weeks of age), grooming test (7–8 weeks of age), open field test (8–9 weeks of age), and elevated plus maze test (9–10 weeks of age).

#### Negative Geotaxis Test

Negative geotaxis was tested on P7, P9, and P11. Each mouse was placed on a board that was tilted at 40°, facing downward. We assessed the latency to turn 180° (i.e., the tip of the nose faced upward). The cutoff time was a maximum of 20 s.

#### Righting Reflex Test

The righting reflex was tested on P7, P9, and P11. Each mouse was placed in the supine position, and the latency to return to the prone position was assessed. The cutoff time was a maximum of 15 s.

#### Cliff Avoidance Test

Cliff avoidance was tested on P7, P9, and P11. Each mouse was set on a desk at a height of 1 m, with its nose positioned outward at the edge of the desk. The latency to avoid the cliff was assessed. The cutoff time was a maximum of 20 s.

#### Hanging Wire Test

The hanging wire test (O'Hara & Co., Tokyo, Japan) was conducted on P25. The mice were placed on a grid wire surface (150 × 150 mm, divided into 10 mm grid squares), and the plane was inverted. The latency to fall was recorded. The cutoff time was a maximum of 600 s.

#### Social Interaction Test

The social interaction test was conducted during both adolescence (5–6 weeks of age) and adulthood (10–11 weeks of age) as previously described (18, 19). For habituation, each mouse was left alone in its home cage in a sound-attenuating chamber for 15 min. One unfamiliar C57BL/6J mouse of the same sex and age was then introduced to the cage. The behavior of the test mouse was video-recorded for 10 min and blindly scored for active social interaction, consisting of sniffing, allo-grooming, mounting, and following. One mouse that went out of its home cage during the 15 min habituation period was excluded from the analysis. Body weight was also recorded when each mouse performed the social interaction test. The number of mice per group was the following: 5–6 weeks of age (*n* = 18 control male mice, *n* = 26 picrotoxin-exposed male mice, *n* = 22 control female mice, *n* = 22 picrotoxin-exposed female mice) and 10–11 weeks of age (*n* = 18 control male mice, *n* = 26 picrotoxin-exposed male mice, *n* = 22 control female mice, *n* = 21 picrotoxin-exposed female mice).

#### Hot Plate Test

The hot plate test (Muromachi, Tokyo, Japan) was conducted at 6–7 weeks of age. Each mouse was set on a hot plate (55.0 ± 0.5°C), and the latency to flicking, jumping, and licking its paws was recorded.

#### Grooming Test

The grooming test was conducted at 7–8 weeks of age and consisted of a 2-day sequence. The first day was the habituation phase and the tested mice were placed in the experimental room for 60 min in their home cage. After 60 min, the mice were placed in a sound-attenuating chamber, and their movements were recorded for 30 min. In this phase, the mice were habituated to the experimental room and apparatus. The second day of the grooming test was the recording day. As on the first day, the mice were habituated to the experimental room, and their movements were recorded in a sound-attenuating chamber for 30 min. Grooming involved wiping the face, nose, ears, and head with forepaws and licking the body other than the face. We counted the number and seconds of grooming for 30 min.

#### Open Field Test

The open field test was conducted at 8–9 weeks of age. The apparatus (Muromachi) consisted of an open field (500 × 500 × 500 mm). Each mouse was placed in the center of the open field and allowed to explore it for 20 min under dim light. Behaviors were automatically recorded by a video tracking system (Muromachi).

#### Elevated Plus Maze Test

The elevated plus maze test (Muromachi) was conducted at 9–10 weeks of age. The apparatus consisted of two closed arms (300 × 60 mm, with 150-mm-high walls) and two open arms (297 × 54 mm). The apparatus was raised 40 cm above the floor. A video tracking system (Muromachi) automatically recorded behaviors.

### RNA Extraction From Whole Brains

We conducted brain collection and RNA extraction according to a previous study (18). After the end of the social interaction test (10–11 weeks of age), whole brains were collected. We examined the whole brain in the present study because the precise brain regions that are associated with ASD have not yet been clearly defined. Total RNA was extracted from the whole brain and homogenized in Ambion TRIzol reagent (Thermo Fisher Scientific, Waltham, MA, USA) using a homogenizer. RNA was isolated using chloroform and precipitated using isopropyl alcohol. The quality of RNA was assessed with Nanodrop 1000 (Thermo Fisher Scientific). All of the RNA samples had an A_260/280_ ratio between 2.01 and 2.02 and A_230/260_ ratio between 2.26 and 2.31.

### Analyses of Whole-Genome Gene Expression

We conducted whole-genome gene expression using a microarray analysis according to a previous study (18). cRNA targets were synthesized and hybridized using the Whole Mouse Genome Microarray according to the manufacturer's instructions (Agilent Technologies, Santa Clara, CA, USA). The array slides were scanned using a SureScan Microarray Scanner (Agilent Technologies, Santa Clara, CA, USA). Before analyzing gene expression, microarray data were normalized and sorted using GeneSpring 14.5 software (Agilent Technologies, Santa Clara, CA, USA). Each sample was normalized by a 75% percentile shift. Compromised probes were removed. Each group comparison was performed using *t*-tests (*p* < 0.05). Each group consisted of five male mice (control mice and picrotoxin-exposed mice). Gene ontology, pathway enrichment analysis, and comparisons with curated studies were conducted using BaseSpace (Illumine, San Diego, CA, USA; https://login.illumina.com/platform-services-manager/?rURL=https://accounts.public. basespace.illumina.com/b/authentication/login.nb;jsessionid=69E78F49406EDD33C6DE7D3C1A2F8FD8&clientId=NBR-Public #/) (20).

### Weighted Gene Co-expression Network Analysis

The WGCNA was performed using the WGCNA package in R software and conducted for step-by-step block-wise network construction and module detection using the package implemented in R version 3.5.2 with the code provided by Langfelder and Horvath (21, 22). To focus on differentially expressed genes, genes with *p* < 0.05 were used for the WGCNA. In accordance with WGCNA default preprocessing steps, any obvious outliers in our sample were checked with an average linkage hierarchical cluster analysis of expression levels. Pearson correlation coefficients for all transcript pairs were then calculated to determine connection strengths between two transcripts. The connection strength between transcript m and transcript n was defined as α_mn_ = [correlation (m, n)], where the β value is set as the weighting coefficient. Power of beta = 30 was chosen based on the scale-free topology criterion (the linear regression model fitting index, *R*^2^, was ~0.9).

### Statistical Analysis

The results of the behavioral tests were analyzed using Prism 9.2.0 software (GraphPad, San Diego, CA, USA). The data were analyzed using unpaired *t*-tests, the Mann-Whitney *U*-test, and two-way repeated-measures analysis of variance (ANOVA) followed by the Bonferroni *post-hoc* test. All of the data are presented as mean ± standard error of the mean (SEM). Values of *p* < 0.05 were considered statistically significant.

## Results

### Effects of Prenatal Exposure to Picrotoxin on Postnatal Development and Motor Function

**Figure 1:**
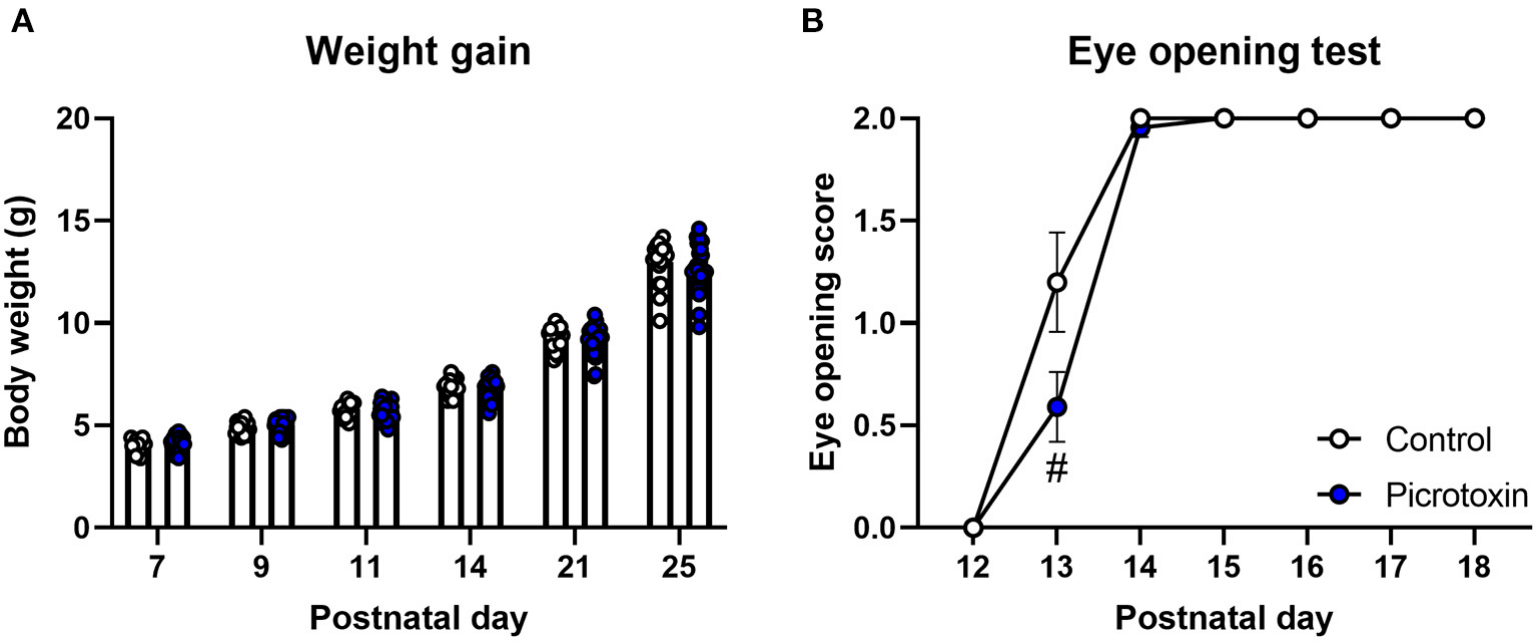
Body maturation and eye-opening scores. **(A)** No significant difference in body weight was found between control and picrotoxin-exposed male mice. **(B)** A trend toward a difference in eye opening was observed between control and picrotoxin-exposed male mice on P13. The data are expressed as mean ± SEM. ^#^*p* < 0.1 [two-way repeated-measures ANOVA in **(A)**, Mann-Whitney *U*-test in **(B)**].

**Figure 2:**
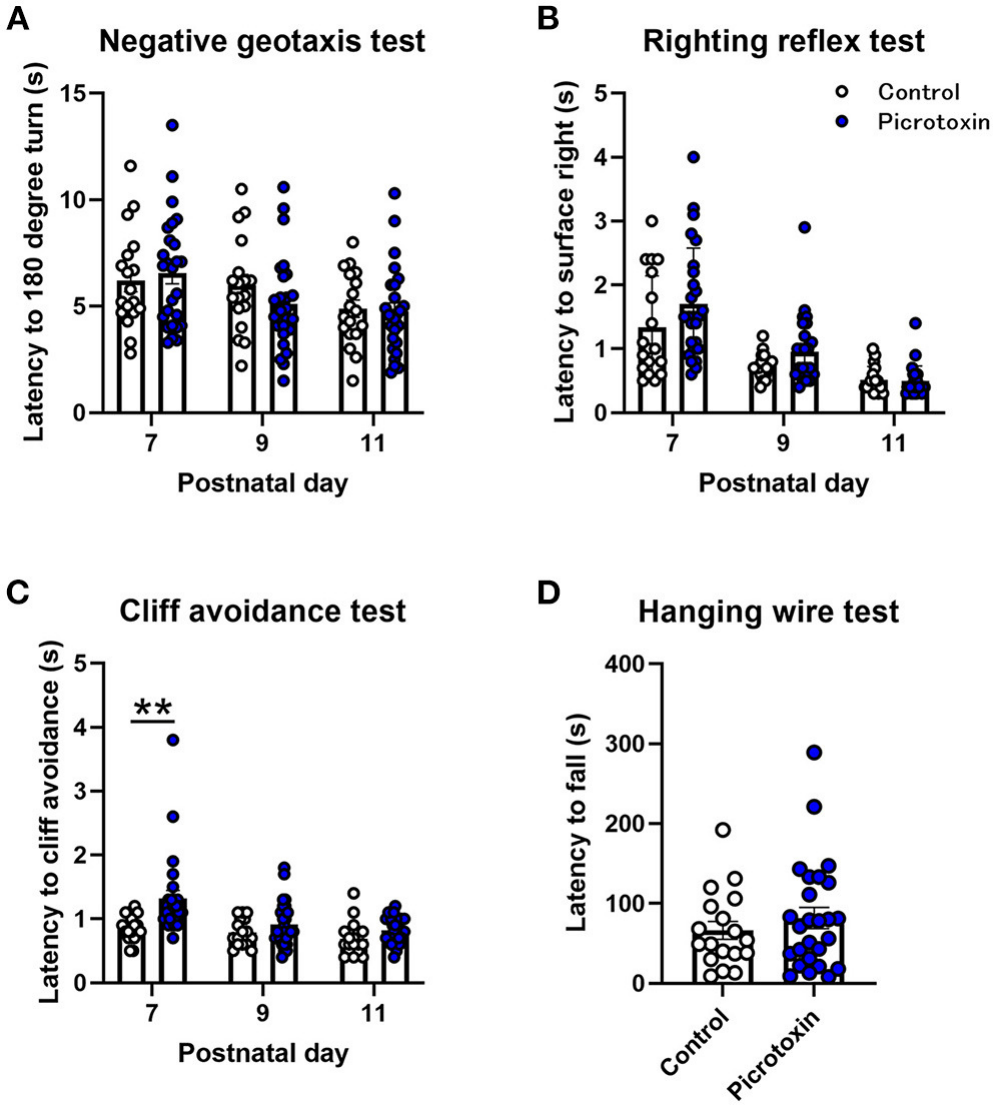
Motor function tests. **(A)** No significant difference in the negative-geotaxis test was found between control and picrotoxin-exposed male mice. **(B)** No significant difference in the righting reflex test was found between control and picrotoxin-exposed male mice. **(C)** Picrotoxin-exposed male mice exhibited a significantly longer latency to cliff avoidance. **(D)** No significant difference in the hanging-wire test was found between control and picrotoxin-exposed mice. The data expressed as mean ± SEM. **p < 0.01 (two-way repeated-measures ANOVA).

No significant difference in body weight was found between control and picrotoxin-exposed male mice from P7 to P25 (Figure 1A). The two-way repeated-measures ANOVA showed no significant main effect of picrotoxin treatment [*F*_(1, 42)_ = 0.281, *p* = 0.599; *n* = 18 control mice, *n* = 26 picrotoxin-exposed mice] and no picrotoxin treatment × day of testing interaction [*F*_(5, 210)_ = 1.035, *p* = 0.398] but a significant effect of day of testing [*F*_(1.916, 80.46)_ = 2,034, *p* < 0.0001]. Eye-opening scores in picrotoxin-exposed male mice tended to be low compared with control male mice on P13 (*U* = 106.500, *p* = 0.059; *n* = 18 control mice, *n* = 22 picrotoxin-exposed mice; Figure 1B), with no significant difference between control and picrotoxin-exposed mice on P12 or P14-18 (*p* > 0.9999). In the negative-geotaxis test, the two-way repeated-measures ANOVA showed no main effect of picrotoxin treatment [*F*_(1, 42)_ = 0.298, *p* = 0.588], no picrotoxin treatment × postnatal day interaction [*F*_(2, 84)_ = 0.805, *p* = 0.450; *n* = 18 control mice, *n* = 26 picrotoxin-exposed mice; Figure 2A], and a significant effect of day of testing [*F*_(1.970, 82.73)_ = 5.361, *p* = 0.007]. In the righting reflex test, the two-way repeated-measures ANOVA showed a trend toward an effect of picrotoxin treatment [*F*_(1, 42)_ = 3.216, *p* = 0.080], no picrotoxin treatment × day of testing interaction [*F*_(2, 84)_ = 1.415, *p* = 0.249; *n* = 18 control mice, *n* = 26 picrotoxin-exposed mice; Figure 2B], and a significant effect of day of testing [*F*_(1.424, 59.80)_ = 39.04, *p* < 0.0001]. In the cliff avoidance test, the two-way repeated-measures ANOVA showed a main effect of picrotoxin treatment [*F*_(1, 42)_ = 12.10, *p* = 0.001], a trend toward a picrotoxin treatment × day of testing interaction [*F*_(2, 84)_ = 3.080, *p* = 0.051; *n* = 18 control mice, *n* = 26 picrotoxin-exposed mice; Figure 2C], and a significant effect of day of testing [*F*_(1.612, 67.72)_ = 10.71, *p* = 0.0003]. The Bonferroni *post-hoc* test revealed a significant difference on P7 (*p* = 0.005). The Bonferroni *post-hoc* test did not reveal a significant difference on P9 (*p* = 0.401) or P11 (*p* = 0.248). No difference in the latency to fall was found between control and picrotoxin-exposed male mice on P25 [*t*_(42)_ = 0.836, *p* = 0.408; *n* = 18 control mice, *n* = 26 picrotoxin-exposed mice; Figure 2D]. The results of these tests indicated that picrotoxin-exposed male mice exhibited a delay in body maturation and poor motor performance compared with control male mice.

### Effects of Prenatal Exposure to Picrotoxin on Affective-Like Behaviors

**Figure 3:**
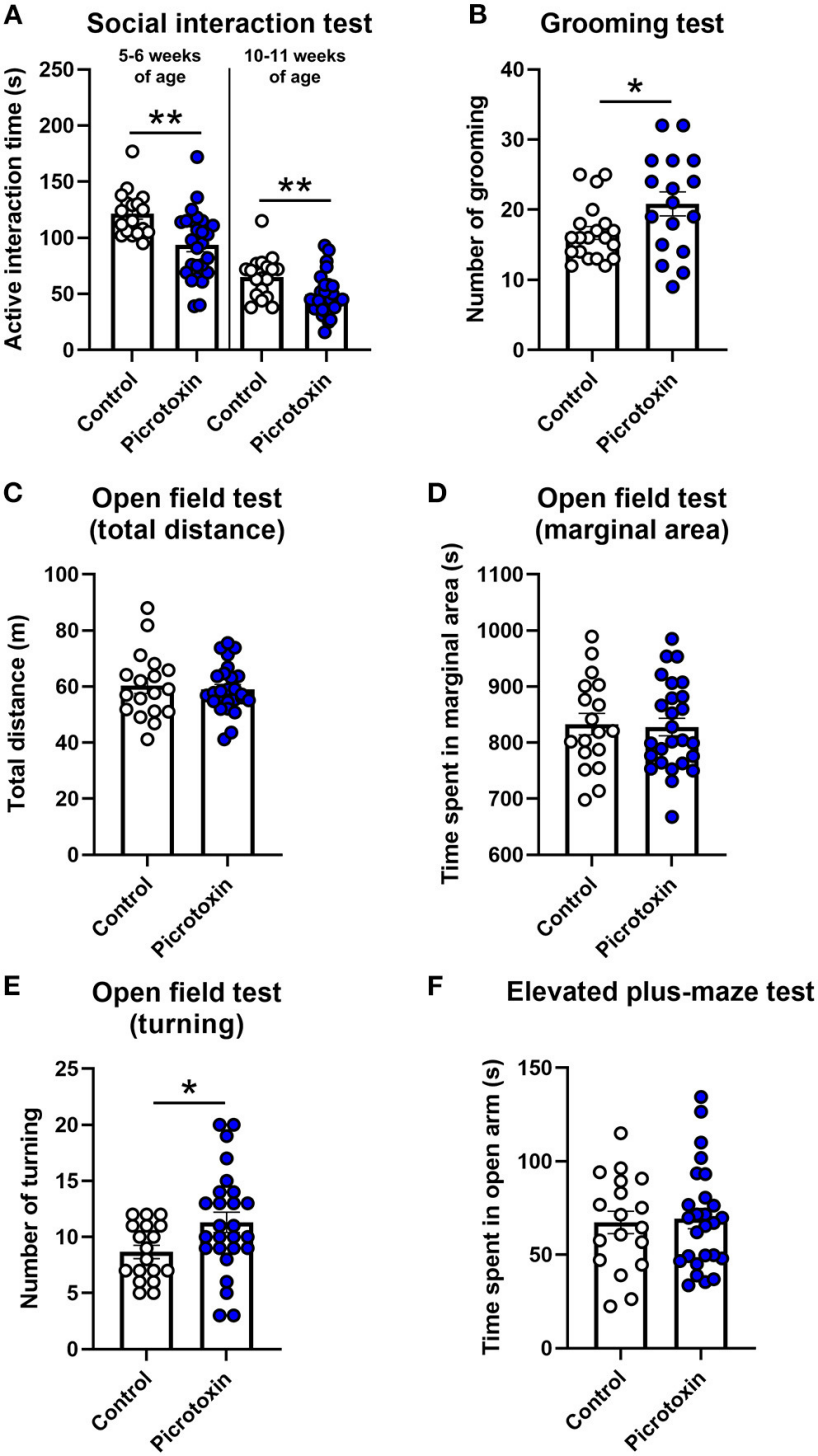
Social interaction, hot plate, grooming, open field, and elevated plus maze tests. **(A)** Social interaction test. Picrotoxin-exposed male mice exhibited a decrease in active social interaction time compared with control male mice at 5–6 and 10–11 weeks of age. **(B)** Grooming test. Picrotoxin-exposed male mice exhibited an increase in the number of grooming episodes compared with control male mice. **(C,D)** Open field test. No significant difference was found between control and picrotoxin-exposed male mice in the total distance traveled **(C)** or time spent in the peripheral area **(D)**. **(E)** Open field test (turning behavior). Picrotoxin-exposed male mice exhibited an increase in the number of turning episodes compared with control male mice. **(F)** Elevated plus maze test. No significant difference in the time spent on the open arms was found between control and picrotoxin-exposed male mice. The data are expressed as mean ± SEM. **p < 0.01, *p < 0.05 (unpaired *t*-test).

We assessed the effects of picrotoxin on social interaction during both 5–6 weeks of age (adolescence) and 10–11 weeks of age (adulthood). In adolescence, picrotoxin-exposed male mice exhibited a decrease in active interaction time compared with control male mice [*t*_(42)_ = 3.378, *p* = 0.002; *n* = 18 control mice, *n* = 26 picrotoxin-exposed mice; Figure 3A]. In adulthood, picrotoxin-exposed male mice exhibited a decrease in active interaction time compared with control male mice [*t*_(42)_ = 2.723, *p* = 0.009; *n* = 18 control mice, *n* = 26 picrotoxin-exposed mice; Figure 3A]. No significant difference in the latency to flicking, jumping, and licking paws in the hot plate test was found between the control and picrotoxin-exposed male mice at 6–7 weeks of age [*t*_(42)_ = 0.547, *p* = 0.587; *n* = 18 control mice, *n* = 26 picrotoxin-exposed mice; Supplementary Figure 2A]. Patients with ASD typically engage in repetitive behaviors (1). The self-grooming test has been used to monitor repetitive behaviors in mouse models of ASD (23). Picrotoxin-exposed male mice exhibited a significant difference in the total number of self-grooming episodes in the grooming test compared with control male mice [*t*_(35)_ = 2.221, *p* = 0.032; *n* = 20 control mice, *n* = 17 picrotoxin-exposed mice; Figure 3B]. No significant difference in the total time of self-grooming in the grooming test was found between control and picrotoxin-exposed male mice [*t*_(35)_ = −1.350, *p* = 0.186; *n* = 20 control mice, *n* = 17 picrotoxin-exposed mice; Supplementary Figure 1B]. No significant difference in the total distance traveled in the open field test was found between control and picrotoxin-exposed male mice [*t*_(42)_ = 0.412, *p* = 0.682; *n* = 18 control mice, *n* = 26 picrotoxin-exposed mice; Figure 3C]. No significant difference in the time spent in the peripheral area was found between control and picrotoxin-exposed male mice [*t*_(42)_ = 0.214, *p* = 0.832; *n* = 18 control mice, *n* = 26 picrotoxin-exposed mice; Figure 3D]. Picrotoxin-exposed male mice exhibited an increase in the number of turning episodes compared with control male mice [*t*_(42)_ = −2.218, *p* = 0.032; *n* = 18 control mice, *n* = 26 picrotoxin-exposed mice; Figure 3E]. No significant difference in the time spent on the open arms of the elevated plus maze was found between control and picrotoxin-exposed male mice [*t*_(42)_ = 0.253, *p* = 0.801; *n* = 18 control mice, *n* = 26 picrotoxin-exposed mice; Figure 3F]. Body weight was recorded at both 5–6 and 10–11 weeks of age in the social interaction test. No significant difference was found between control and picrotoxin-exposed male mice in either adolescence or adulthood (Supplementary Results and Supplementary Table 1). After the social interaction test at 10–11 weeks of age, we collected the whole mouse brain and recorded brain weights to confirm the effects of picrotoxin treatment. No significant difference in brain weight was found between control and picrotoxin-exposed male mice (Supplementary Results and Supplementary Table 1).

We also analyzed body maturation, motor function, social interaction, behavior in the hot plate test, grooming behavior, activity in the open field test, and anxiety-like behavior in the elevated plus maze test in control female mice and picrotoxin-exposed female mice. No significant difference was found between control and picrotoxin-exposed female mice in the social interaction test. All behavioral data in female mice are presented in the Supplementary Results and Supplementary Table 2.

### Effects of Picrotoxin Exposure *in utero* on Gene Expression

Picrotoxin-exposed male mice (*n* = 5) exhibited the differential expression of 465 genes (438 upregulated genes, 27 downregulated genes) compared with control male mice (*n* = 5; Table 1). To further analyze the functional significance of these genes, enrichment pathway analysis was performed for each upregulated and downregulated gene using BaseSpace. The enrichment pathways for each regulated gene are described in Table 2, Supplementary Tables 4, 5. The top five pathways for upregulated genes were odorant binding, neuropeptide receptor activity, positive regulation of neutrophil migration, coenzyme A (CoA)-ligase activity, and acid-thiol ligase activity (Table 2). The top five pathways for downregulated genes were protein N-terminus binding, regulation of myeloid cell differentiation, carbohydrate binding, regulation of hemopoiesis, and negative regulation of hemopoiesis (Table 2). We also identified common genes whose expression was altered in whole brains between picrotoxin-exposed male mice and VPA-exposed male mice [which have been used as an animal model of ASD and were reported in our previous study (18)]. The common genes whose expression was altered were *Camk1d, Platr26, Zfp599, Fyb*, and *Cdc7* (Table 3). These genes were upregulated in both picrotoxin-exposed male mice and VPA-exposed male mice. Of these altered genes, *Fyb* expression was recovered by rapamycin treatment in VPA-exposed mice (18). These results suggest that exposure to picrotoxin *in utero* alters gene expression in the offspring brain, and the genes whose expression is altered are common to an existing mouse model of ASD.

**Table 1:** Number of genes whose expression was altered by picrotoxin treatment.

**Group**	**Total altered genes**	**Up regulated genes**	**Down regulated genes**
Picro/Control	465	438	27

*Picro, picrotoxin treatment*.

**Table 2:** Gene ontology pathway enrichment analysis of upregulated and downregulated genes.

**Biogroups**	**Normalized**	***P*-value**	**Common**
	**score**		**genes**
**Up regulated genes**			
Odorant binding	100	0.0002	12
Neuropeptide receptor activity	73	0.002	4
Positive regulation of neutrophil migration	70	0.0025	3
CoA-ligase activity	67	0.0032	2
Acid-thiol ligase activity	65	0.0038	2
**Down regulated genes**			
Protein-N-terminus binding	100	0.0003	3
Regulation of myeloid cell differentiation	94	0.0005	3
Carbohydrate binding	77	0.002	3
Regulation of hemopoiesis	77	0.0027	3
Native regulation of hemopoiesis	65	0.005	2

**Table 3:** Genes whose expression was altered in picrotoxin-exposed and VPA-exposed mice.

**Gene**	**Fold change**,	**Fold change**,
	***P*-value (Picro)**	***P*-value (VPA)**
*Camk1d*	1.3145 (0.0101)	1.4299 (9.80-E04)
*Platr26*	1.2708 (0.0165)	1.5662 (0.0133)
*Zfp599*	1.2675 (0.0454)	1.2655 (0.0262)
*Fyb*	1.2231 (0.0474)	2.7767 (4.88-E04)
*Cdc7*	1.2114 (0.0259)	1.3891 (0.0396)

*Picro, picrotoxin; VPA, valproic acid*.

### Weighted Gene Co-expression Network Analysis

**Figure 4:**
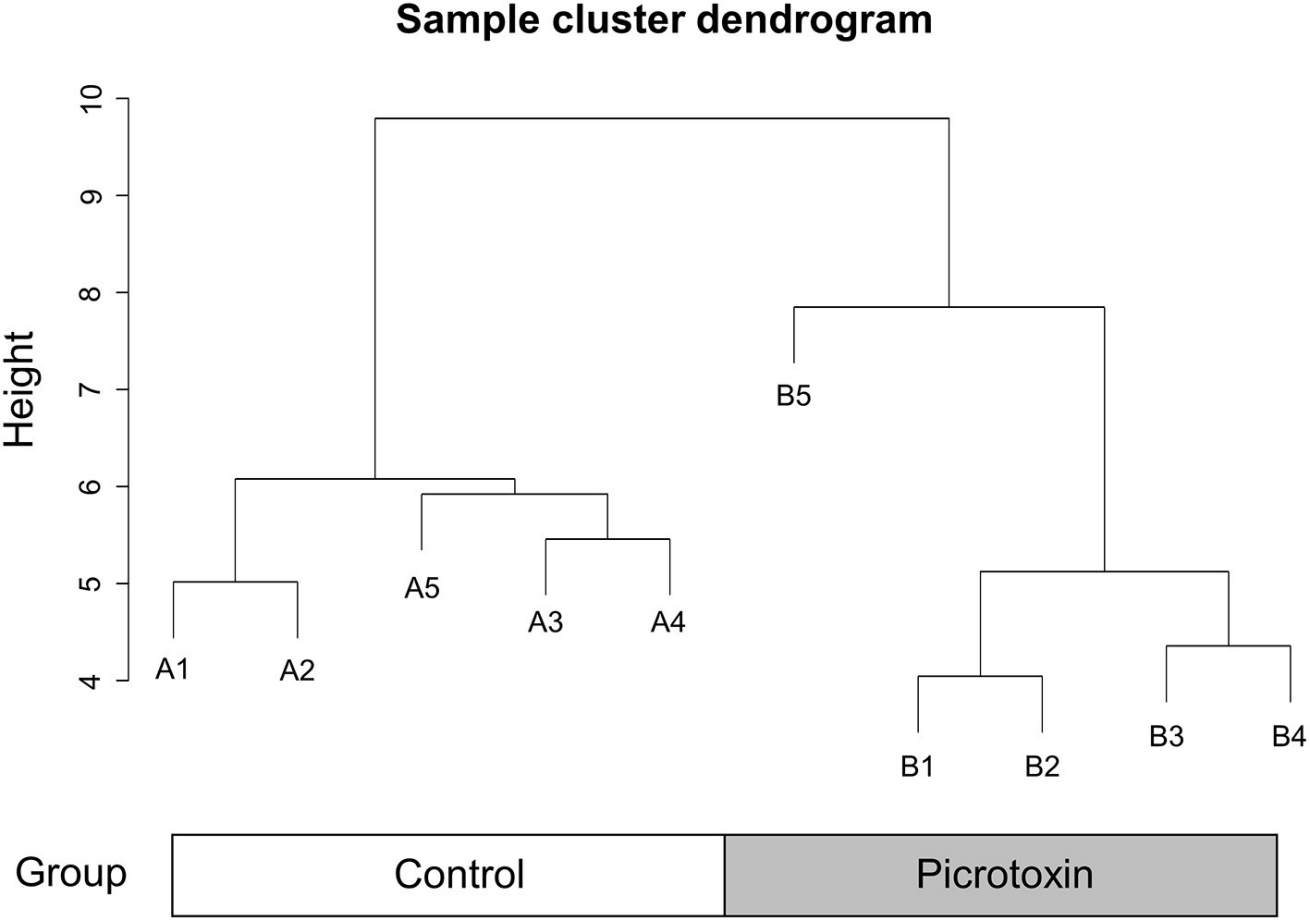
Sample cluster dendrogram. Hierarchical clustering of samples of 1,867 genes in the mouse whole brain.

**Figure 5:**
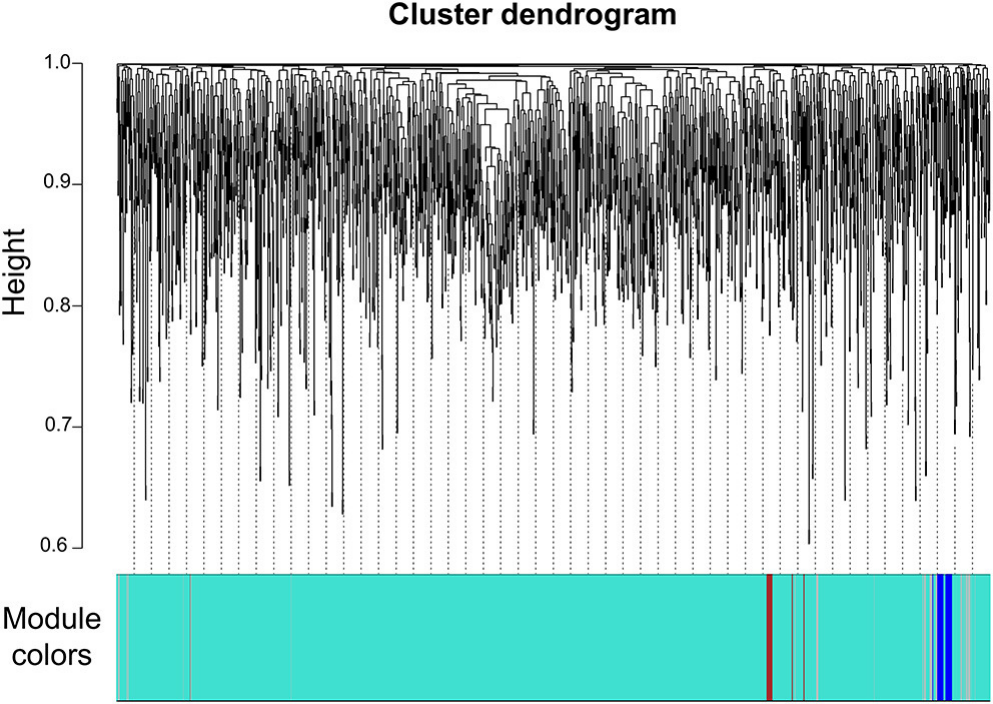
Gene dendrogram. Gene dendrogram and clustered modules coded by four colors.

**Figure 6:**
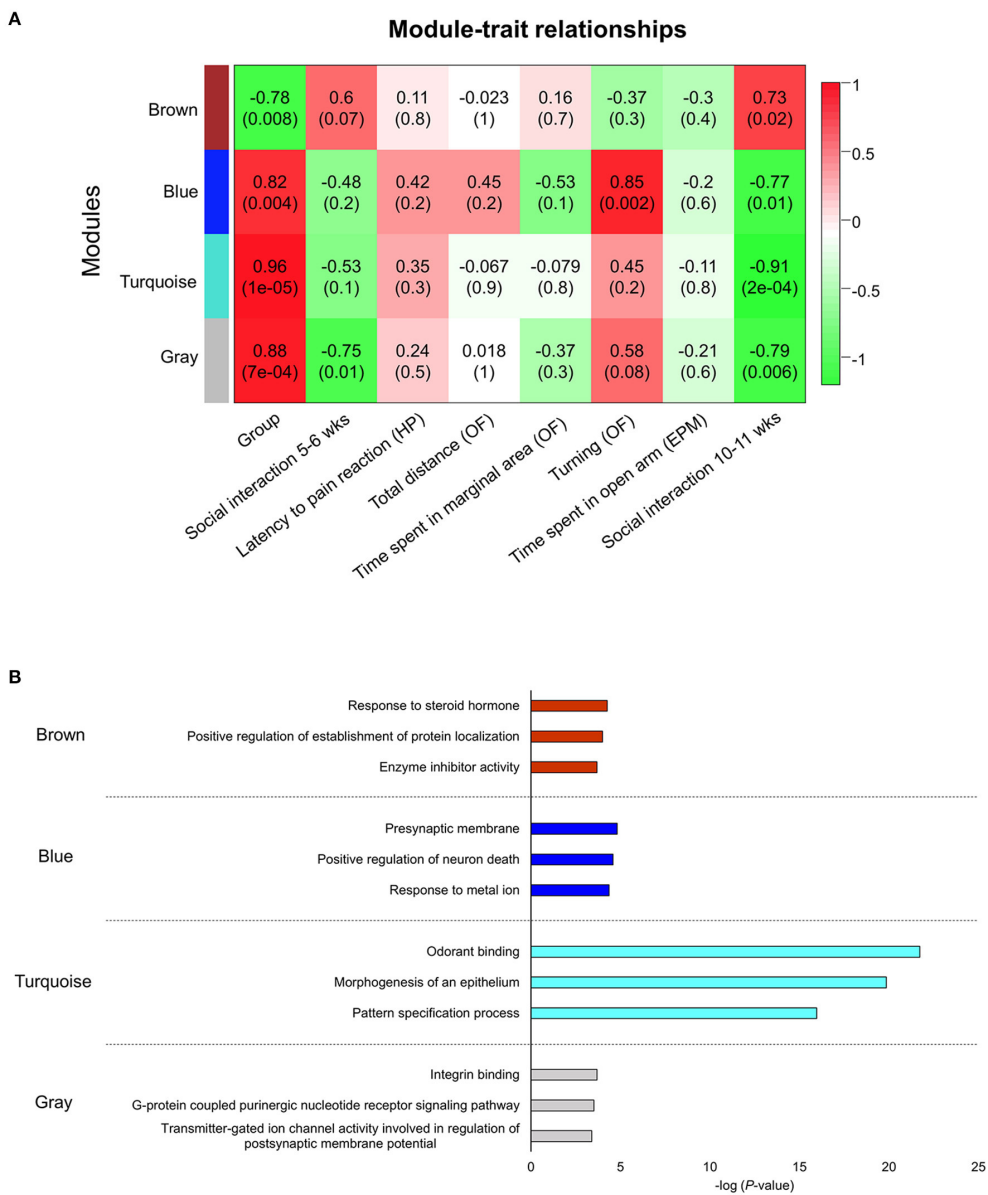
Network construction of co-expressed genes that correlated with performance in the behavioral tests and gene ontology pathway enrichment analysis of each module. **(A)** Module trait correlation for treatment, social interaction (5–6 weeks of age), latency to flicking, jumping, licking paws (hot plate test), total distance traveled (open field test), time spent in the peripheral area (open field test), turning episodes (open field test), time spent on the open arms (elevated plus maze test), and social interaction (10–11 weeks of age). HP, hot plate test; OF, open field test; EPM, elevated plus maze test. **(B)** The blue module was the most correlated with turning episodes in the open field test. The turquoise module was the most correlated with social interaction (10–11 weeks of age).

We next constructed a WGCNA to explore relationships between behavioral and gene traits in picrotoxin-exposed mice. The dendrogram represents a single tight-clustering branch of each sample (Figure 4). Gene expression in the whole brain in both control and picrotoxin-exposed male mice was discriminated into two clusters, indicating that there were no outliers in the samples. A total of four distinct gene modules were identified from the expression of 1,867 genes using a dynamic tree cutting algorithm (Figure 5). The brown, blue, turquoise, and gray gene modules included 18, 28, 1,776, and 45 genes, respectively. The 45 uncorrelated genes were grouped into in the gray module. To find modules of interest, correlations between the color modules and performance in the behavioral tests were computed (Figure 6A). Performance in the grooming test was excluded in this correlation analysis because we could not find a consistent result with regard to the number and time of self-grooming. Picrotoxin treatment significantly correlated with four modules (brown module: *r* = −0.78, *p* = 0.008; blue module: *r* = 0.82, *p* = 0.004; turquoise module: *r* = 0.96, *p* = 1e-05; gray module: *r* = 0.88, *p* = 7e-04). Performance in the social interaction test at 5–6 weeks of age was significantly associated with picrotoxin treatment (gray module: *r* = −0.75, *p* = 0.01). All four modules significantly correlated with performance in the social interaction test at 10–11 weeks of age. Among these four modules, the turquoise module showed the highest negative correlation (brown module: *r* = 0.73, *p* = 0.02; blue module: *r* = −0.77, *p* = 0.11; turquoise module: *r* = −0.91, *p* = 2e-04; gray module: *r* = −0.79, *p* = 0.006). The number of turning episodes in the open field test was significantly associated with the blue module (*r* = 0.85, *p* = 0.002). The enrichment pathways for each module are listed in Supplementary Tables 6–9. The top three pathways for each module are described in Figure 6B. The turquoise module that was most associated with performance in the social interaction test at 10–11 weeks of age was enriched for the odorant binding and tissue differentiation and development pathways (Figure 6B, Supplementary Table 6). The blue module that was most associated with performance in the open field test at 8–9 weeks of age was enriched for synapse-associated pathways (Figure 6B, Supplementary Table 7).

## Discussion

In the present study, we found that prenatal exposure to picrotoxin in mice on day 12.5 of gestation had long-term and selective effects on postnatal behaviors and gene expression in male offspring. In the behavioral tests, prenatal exposure to picrotoxin (i) tended to delay body maturation and motor function, (ii) induce impairments in social interaction in both adolescence and adulthood, and (iii) increase the number of turning episodes in the open field test. The gene expression analysis showed that picrotoxin-exposed mice exhibited alterations of the expression of 465 genes, including five genes that were in common with alterations in VPA-exposed male mice. The WGCNA showed that social interaction in adulthood had the strongest negative correlation with the turquoise gene module, and turning episodes in the open field test had a strongest positive correlation with the blue gene module. “Odorant binding” and “presynaptic membrane” were the top pathways for the turquoise and blue gene modules, respectively.

The pathophysiology of ASD has not been well-defined. Many hypotheses suggest causal explanations of ASD. An E/I imbalance in the mature brain is theorized to be a key pathophysiological mechanism of ASD (24–28). The influence of GABA during prenatal development is excitatory, and GABA/GABA_A_ receptor signaling plays a key role in cellular processes during development. Previous studies reported that disturbances of GABA/GABA_A_ receptor signaling affect postnatal behaviors in offspring. Perinatal exposure to picrotoxin in rat dams affected sexual behavior in male offspring by increasing the latency to the first mount and intromission (12). The neonatal blockade of GABA_A_ receptors with bicuculline produced abnormal passive avoidance memory and increased brain-derived neurotrophic factor levels in the brain on P61-70 (11). Prenatal exposure to ethanol, a GABA_A_ receptor agonist, dysregulated the vertical and horizontal cleavage planes of neural progenitors in the developing neocortex, and picrotoxin treatment prior to ethanol administration restored regulation of the cleavage plane to control levels (16). Tyzio et al. reported that a rat model of VPA-induced ASD and mouse model of Fragile X syndrome abolished the switch of GABA from excitatory to inhibitory transmission. Maternal pretreatment with bumetanide, an antagonist of Na-K-Cl cotransporter 1 chloride importers, before delivery improved this switch in GABA activity and aberrant ultrasonic vocalizations in offspring (29). Tochitani et al. recently reported that the modulation of GABA_A_ receptors by agonists and antagonists did not cause social deficits but resulted in the rapid loss of interest in a stranger mouse compared with control mice (15). The different findings between their study and the present study might be attributable to the treatment duration, dose, social interaction test that was conducted, or mouse strain. Nonetheless, the present results support the findings of Tochitani et al. and further our understanding of correlations between disturbances of GABA_A_ receptor signaling during the embryonic period and the pathophysiology of ASD. Tochitani et al. also reported that the administration of 5.0 mg/kg picrotoxin twice daily on embryonic days 10–12 affected neuronal progenitor cells in the neocortex on embryonic day 13, decreased Tbr2-positive cells, increased Pax 6-positive cells, and decreased Doublecortin (Dcx; a marker for immature and migrating neurons)-positive layers (15). Picrotoxin administration during the embryonic period disturbs development of the neocortex and might contribute to the pathophysiology of ASD. In the present study, we found that prenatal GABA_A_ receptor blockade with picrotoxin affected behavior in male offspring in both adolescence and adulthood and caused ASD-like behaviors, including deficits of social interaction.

Picrotoxin-exposed male mice exhibited alterations of genes, including 438 upregulated genes and 27 downregulated genes, compared with control male mice. We conducted gene ontology pathway enrichment analysis to understand the function of these genes. We found that genes whose expression was upregulated are related to odorant binding, neuropeptide receptor activity, neutrophil migration, and catalysis (Table 2). The downregulated genes are related to protein-N-terminus binding, myeloid cell differentiation, carbohydrate binding, and hemopoiesis (Table 2). Upregulated and downregulated genes with normalized scores of 100 were related to odorant binding and protein-N-terminus binding, respectively. A previous clinical study reported that patients with ASD exhibited aberrant olfactory responses, with a lower discrimination score and higher bias score in an olfactory test (30). One of the identified genes in the protein-N-terminus binding pathway was *Hesx1*, which is required to program human embryonic stem cell neural fate (31).

We also detected alterations of the expression of genes that were regulated in the same direction (i.e., upregulated) in picrotoxin-exposed male mice and VPA-exposed male mice. Exposure to VPA has been used as an animal model of ASD (18, 32–34). We detected five genes whose expression was commonly altered between picrotoxin-exposed male mice and VPA-exposed male mice: *Camk1d, Platr26, Zfp599, Fyb*, and *Cdc7* (Table 3). *Camk1d* and *Cdc7* were reportedly associated with brain and embryonic development (35, 36). *Platr26* is a long non-coding RNA, and *Platr1*-*32* were suggested to be functionally integrated into the mouse embryonic stem cell gene expression program (37). Zinc finger protein plays a key role in tissue development and differentiation (38). *De novo* deletions in the 19q.13.11 region that encompasses four KRAB-ZNFs, including *Zfp* 599, were identified in two unrelated cases of microcephaly (39). In our previous study (18), *Fyb* expression was recovered by the mammalian target of rapamycin complex 1 inhibitor rapamycin in VPA-exposed male mice. *Fyb* is broadly expressed in the mouse brain and strongly expressed in the olfactory bulb (40). Valproic acid is clinically used as an anti-epileptic drug and increases GABA release (41). We speculate that these five genes are key molecules in the pathophysiology of ASD-like behavior in mice, in which GABA signaling is disturbed during embryonic periods.

A WGCNA was used to detect correlations between co-expression genes and performance in various behavioral tests. We identified four modules based on the WGCNA. All four gene modules (brown, blue, turquoise, and gray) had a significant correlation with picrotoxin treatment (Group) and social interaction at 10–11 weeks of age, and the blue gene module had a significant correlation with turning episodes in the open field test. The turquoise module had the strongest correlation with performance in the social interaction test at 10–11 weeks of age. The top three enrichment pathways for the turquoise module were odorant binding, epithelium morphogenesis, and pattern specification process. Overall, the turquoise module included pathways that are related to tissue differentiation and development (Figure 6B, Supplementary Table 6). Interestingly, the most significantly enriched pathway was “odorant binding.” Active interaction time in the social interaction test consisted of sniffing, allo-grooming, mounting, and following. The sniffing ratio was the most active social interaction behavior (Supplementary Table 3). Aberrant olfactory function may have altered social interaction in picrotoxin-exposed male mice. Patients with ASD also exhibit sensory issues, including aberrant olfactory function (42). A previous clinical study reported that patients with ASD exhibited aberrant odor awareness (43). A significant correlation was found between olfactory threshold and social problems, determined by the Child Behavior Checklist, in male children with ASD (44). The sniffing duration ratio correlated with the social affect component of the Autism Diagnostic Observation Schedule but not the restricted/repetitive behavior component (45). Impairments in social interaction in ASD are often caused by the misreading of emotional cues. Endevelt-Shapira et al. investigated social chemosignals in adult patients with ASD. When typically developing participants sniffed an undetectable scent of fear (i.e., skydiver sweat) or control sweat, they presented enhanced autonomic arousal responses (i.e., electrodermal activity) in response to the skydiver sweat compared with control sweat, whereas patients with ASD did not exhibit such changes in the autonomic arousal response (46). These clinical findings suggest that the olfactory system plays a key role in sociability in patients with ASD. Franco et al. reported that a reduction of lateral inhibition in the brain impaired odor discrimination and social behavior in a *Drosophila* model of Fragile X syndrome that exhibited ASD-like behaviors (47). *Scn*1*a*^+^/− mice also exhibit ASD-like behaviors, including social deficits and the avoidance of social odors (e.g., male urine odors), in a Y-maze olfactory choice test (48). Mitral cells in the olfactory bulb are generated on embryonic days 9–13. The olfactory tubercle receives a robust axonal projection from mitral cells that are generated on embryonic day 12 (49). The present study did not investigate specific changes in the olfactory bulb or olfactory cortex, but our findings suggest that picrotoxin administration during the embryonic period may affect development of the olfactory system and its connections with other brain regions. The blue module was directory correlated with turning behavior in the open field test at 8–9 weeks of age. The blue module included pathways that are related to synapses as the most enriched pathways (Figure 6B, Supplementary Table 7). The top network in the blue module was “presynaptic membrane.” Presynaptic function has been investigated in neurodevelopmental disorders, including ASD (50). Patients with ASD and animal models of ASD exhibit different behaviors in a novel environment. Patients with ASD exhibited higher stress responses to novel stimuli compared with controls (51, 52). The ablation of metabotropic glutamate receptor 5 in mice resulted in synaptic deficiency and an increase in novelty-induced locomotion compared with wildtype mice (53). We speculate that the high number of turning episodes is an aberrant response to a novel environment. Patients with ASD often exhibit stimming behavior (e.g., hand-flapping, body rocking, and spinning in circles), also known as repetitive/restricted behavior, to manage their emotions and overwhelming sensory inputs (54). A previous study reported that prenatal zinc deficiency in mice, which is an animal model of ASD, resulted in a side preference of rotational behavior in a round arena (a 360° turn was considered a rotation) (55), but we did not observe differences between right and left turning episodes in the present study (P8, Supplementary Table 10).

In the present study, the detected pathways in the turquoise and blue modules are helpful for clarifying the mechanisms of ASD-like behaviors, which may aid the identification of possible treatment targets. The uncorrelated genes were assigned to the gray module and was thus normally excluded from further analysis. However, the gray module negatively correlated with performance in the social interaction test in both adolescence and adulthood in picrotoxin-exposed male mice. The key pathways that caused social deficits in both adolescence and adulthood were in the gray module. Social interaction at 10–11 weeks of age was the most correlated with the turquoise module. Social interaction at 5–6 weeks of age was not correlated with the turquoise module. We speculate that adults and adolescents may recruit different pathways that contribute to social behaviors. The present findings may help clarify differences in neural mechanisms that are involved in sociability between adults and adolescents.

We observed sex differences in picrotoxin-exposed mice. Exposure to picrotoxin during the embryonic period caused social deficits in males but not females. The ratio of males to females is higher in the ASD patient population (2). A previous study reported that treatment with the GABA_A_ receptor inhibitor bicuculine during the neonatal period elicited aberrant anxiety-like behavior in male mice but not in female mice (10, 11). Tracosis et al. reported that the autistic male mice showed reduced the Hurst exponent in resting state fMRI in the medial prefrontal cortex, indicating increased excitation but female mice did not show the reduced the Hurst exponent (56). Valproic acid-exposed female mice did not exhibit impairments in social interaction (33) but exhibited a decrease in Nissl-positive cells in the somatosensory cortex compared with male mice (57). Female mice also did not exhibit ASD-like behaviors despite having the Nlgn3/Cyfip1 risk allele (58). A recent study reported that low placenta levels of allopregnanolone (ALLO), a progesterone-derived GABA_A_ receptor modulator, resulted in sex-dependent alterations of neurodevelopment and behavior in offspring (59). Male plKO mice that were derived from pregnant plKO mice that have specific placental ALLO reduction exhibited ASD-like behaviors, including social deficits, and an increase in cerebellar myelin proteins on P30, whereas female plKO mice did not exhibit these changes (59). Similar results were found in human preterm infants that were characterized by premature loss of the placenta (59). This study suggests that GABA_A_ receptor function in female mice might not be disrupted by low ALLO levels or that female mice may recruit a compensatory mechanism in response to impairments in GABA_A_ receptor function (59). Sex bias in ASD may be related to chromosomal and hormonal mechanisms, immune activation, or interactions among sex, genetics, and environmental factors (60). These previous studies and the present study suggest that female sex may be a protective factor against ASD, making males more susceptible to ASD. Further studies are warranted to verify possible sex differences in animal models of ASD.

We suggest that exposure to picrotoxin during the embryonic period contributes to the pathophysiology of ASD via GABA_A_ receptor signaling. Picrotoxin also blocks homomeric glycine receptor (GlyR) subtypes (61, 62). Glycine receptors are ligand-gated chloride channels that are expressed in the brain and spinal cord (63). Pilorge et al. reported that male patients with ASD had a *de novo* missense mutation of *GLRA2*, which encodes the GlyRα2 subunit. These authors also found that *Glra2* knockout mice did not exhibit social behavior and spent less time exploring a novel object (64). In brain slices that were prepared on embryonic day 11, pretreatment with picrotoxin inhibited the response of neuronal progenitors to the application of taurine, which is a ligand of GlyRs and GABA_A_ receptors (15). We did not investigate the specific mechanisms of action of picrotoxin during the embryonic period in the present study, but GABA_A_ receptor or GlyR inhibition by picrotoxin is suggested to contribute to the pathophysiology of ASD.

The present study found ASD-like behaviors, gene aberrations, and correlations between gene expression and behavior in mice that were exposed to picrotoxin during the embryonic period. Our results provide a better understanding of the pathophysiology of ASD. Our study suggests that prenatal exposure to an GABA_A_ receptor inhibitor induces ASD-like behaviors in offspring. The prenatal inhibition of GABA_A_ signaling may a mechanism that contributes to ASD.

## Data Availability Statement

The original contributions presented in the study are included in the article/Supplementary Materials, further inquiries can be directed to the corresponding author/s.

## Ethics Statement

The animal study was reviewed and approved by Animal Experimentation Ethics Committee of Tokyo Metropolitan Institute of Medical Science.

## Author Contributions

HK-M and KI designed the experiments and wrote the paper. HK-M performed the mouse behavioral testing and analyzed the behavioral data. HK-M and HH analyzed the gene expression data. HH conducted the WGCNA and wrote the methods of WGCNA. YN and YT performed the microarray analysis. HK-M and YH generated the picrotoxin mouse model of ASD and were responsible for breeding management. AS, MT, YK, SU, and TM participated in refinements of the experiments and discussion. All authors read and approved the final manuscript to be published.

## Funding

This research was supported by Grants-in-Aid for Scientific Research from the Japan Society for the Promotion of Science, KAKENHI [17K15765, JP16H06276 (AdAMS)].

## Conflict of Interest

The authors declare that the research was conducted in the absence of any commercial or financial relationships that could be construed as a potential conflict of interest.

## Publisher's Note

All claims expressed in this article are solely those of the authors and do not necessarily represent those of their affiliated organizations, or those of the publisher, the editors and the reviewers. Any product that may be evaluated in this article, or claim that may be made by its manufacturer, is not guaranteed or endorsed by the publisher.
