# Supplementary material for: Exposure to GABAA Receptor Antagonist Picrotoxin in Pregnant Mice Causes Autism-Like Behaviors and Aberrant Gene Expression in Offspring
Source: Front Psychiatry. 2022 Feb 3;13:821354. doi: 10.3389/fpsyt.2022.821354 (PMC8850354; doi:10.3389/fpsyt.2022.821354)
Supplement: Supplementary file 1 [file Data_Sheet_1.PDF]

Supplementary Materials

## Supplementary Methods, Results, Figures, and Tables

### Determination of picrotoxin dose

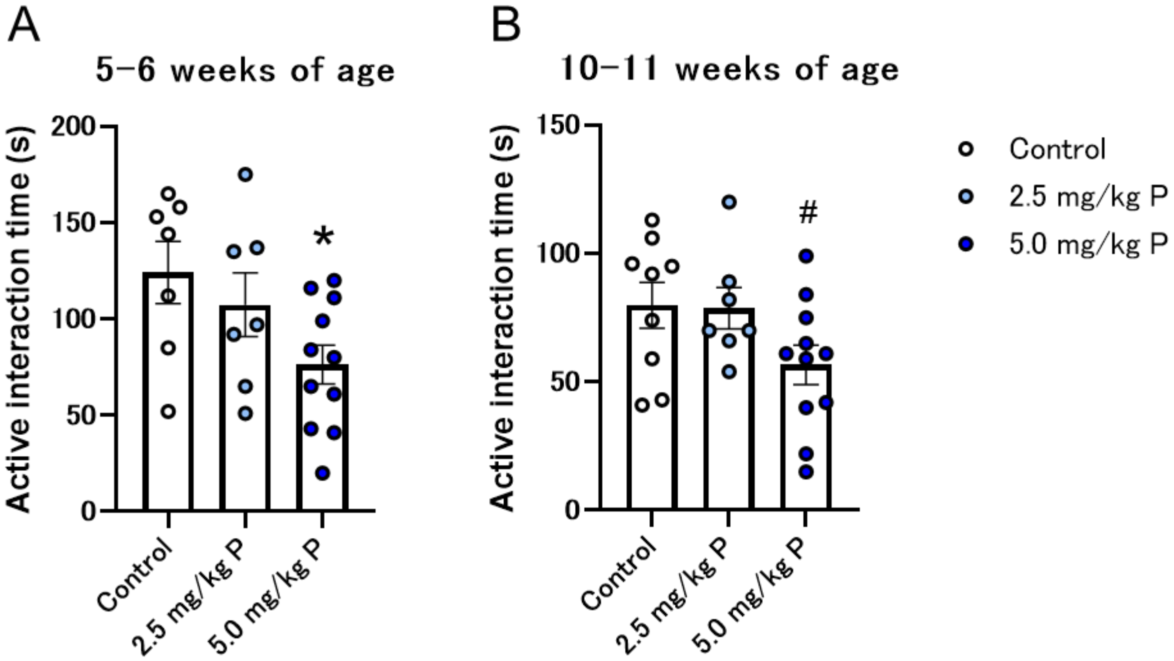


Figure S1. Performance in the social interaction test in male offspring from female mice that were treated with 2.5 and 5.0 mg/kg picrotoxin. Pregnant female mice received 2.5 or 5.0 mg/kg picrotoxin on gestation day 12.5. (A) Male mice that were born to mothers that received 2.5 mg/kg picrotoxin did not exhibit impairments in social interaction at 5-6 weeks of age ($t_{12}$ = 0.723, $p$ = 0.484; $n$ = 7 control mice, $n$ = 7 mice that were exposed to 2.5 mg/kg picrotoxin), whereas male offspring that were exposed to 5.0 mg/kg picrotoxin exhibited a decrease in active interaction time ($t_{16}$ = 2.659, $p$ = 0.017; $n$ = 7 control mice, $n$ = 11 mice that were exposed to 5.0 mg/kg picrotoxin). (B) Similar results were found at 10-11 weeks of age ($t_{14}$ = 0.095, $p$ = 0.926, $n$ = 9 control mice, $n$ = 7 mice that were exposed to 2.5 mg/kg picrotoxin; $t_{18}$ = 1.989, $p$ = 0.0621, $n$ = 9 control mice, $n$ = 11 mice that were exposed to 5.0

mg/kg picrotoxin). Based on these results, we tested the 5.0 mg/kg picrotoxin dose in the present study. *$p < 0.05$, #$p < 0.1$ (unpaired *t*-test). 2.5 mg/kg P, 2.5 mg/kg picrotoxin-exposed male mice; 5.0 mg/kg P, 5.0 mg/kg picrotoxin-exposed male mice.

**Results of the hot plate and grooming tests (related to Fig. 3 in main text)**

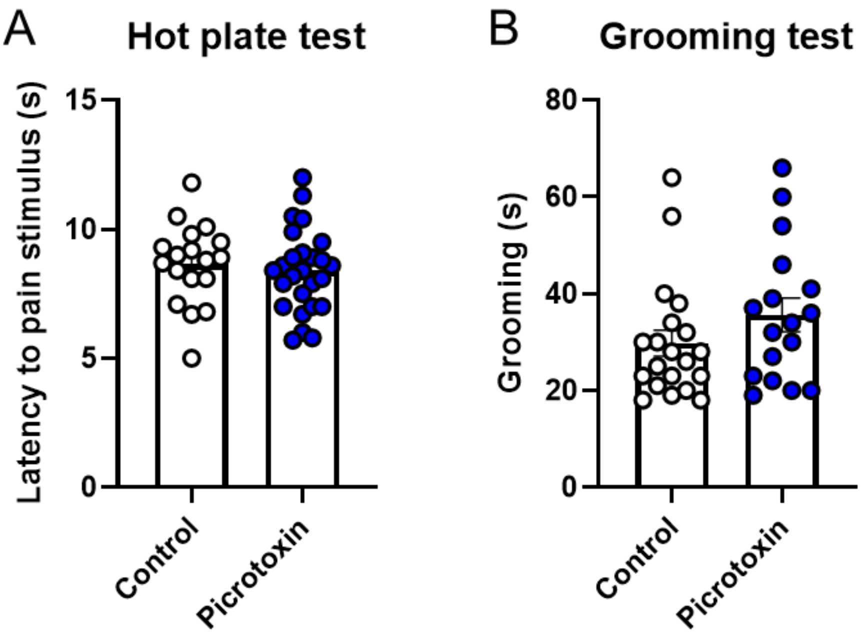


Figure S2. Hot plate test and grooming test in male offspring. (A) No significant difference between control and picrotoxin-exposed mice in the hot plate test. (B) No significant difference in grooming time between control and picrotoxin-exposed mice. The data were analyzed using unpaired *t*-test.

**Body and brain weights in male offspring**

Body weight was recorded immediately before the social interaction test at both 5-6 and 10-11 weeks of age. No significant difference was found between control and picrotoxin-exposed male mice in adolescence (5-6 weeks of age; $t_{42} = 1.558$, $p = 0.127$; Table S1). In

adulthood, no significant difference was found between control and picrotoxin-exposed male mice (10-11 weeks of age; $t_{42} = 0.873$, $p = 0.388$; Table S1). After the social interaction test at 10-11 weeks of age, we collected whole mouse brains and assessed brain weights. No significant difference was found in brain weight between control and picrotoxin-exposed male mice ($t_{42} = 0.095$, $p = 0.925$; Table S1).

Table S1. Body and brain weights in control and picrotoxin-exposed male mice.

| | **Control mice** | **Picrotoxin-exposed mice** |
|---|---|---|
| **Body weight, 5-6 weeks of age (g)** | 21.3 (0.27) | 20.6 (0.27) |
| **Body weight, 10-11 weeks of age (g)** | 26.6 (0.39) | 26.2 (0.36) |
| **Brain weight (mg)** | 467.6 (2.44) | 467.9 (2.36) |

The data in parentheses are standard errors of the mean (SEMs).

## Results of behavioral tests in female offspring

*Effects of prenatal exposure to picrotoxin on postnatal development and motor function*

The behavioral data are presented in Table S2. The two-way repeated-measures ANOVA showed no significant effect of group on body weight ($F_{1,42} = 1.718$, $p = 0.197$) but a significant picrotoxin treatment × postnatal day interaction ($F_{5,210} = 3.315$, $p = 0.010$). Both treatment groups (i.e., picrotoxin-exposed and saline-exposed) exhibited a progressive increase in body weight on P7-25 ($F_{2.129,89.43} = 2109$, $p < 0.0001$). No significant difference was found in eye opening scores on P12 ($U = 242$, $p > 0.999$; $n = 22$ control mice; $n = 21$ picrotoxin-exposed mice), with no significant difference between control and picrotoxin-exposed female mice on P13 ($p = 0.302$), P14 ($p = 0.607$), or P15-P18 ($p > 0.999$). In the

negative-geotaxis test, the two-way repeated-measures ANOVA showed no main effect of picrotoxin treatment ($F_{1,42}$ = 0.107, $p$ = 0.745) and no picrotoxin treatment × postnatal day interaction ($F_{2,84}$ = 0.722, $p$ = 0.489; $n$ = 22 control mice; $n$ = 22 picrotoxin-exposed mice). A trend toward an effect of postnatal day was observed ($F_{1.942,81.57}$ = 2.673, $p$ = 0.077). In the righting reflex test, the two-way repeated-measures ANOVA showed no main effect of picrotoxin treatment ($F_{1,42}$ = 0.080, $p$ = 0.779) and no picrotoxin treatment × postnatal day interaction ($F_{2,84}$ = 0.007, $p$ = 0.993; $n$ = 22 control mice; $n$ = 22 picrotoxin-exposed mice). Both treatment groups exhibited a progressive decrease in the latency to right on P7, P9, and P11 ($F_{1.181,49.58}$ = 32.29, $p$ < 0.0001). In the cliff avoidance test, the two-way repeated-measures ANOVA showed a main effect of picrotoxin treatment ($F_{1,42}$ = 4.664, $p$ = 0.037) but no picrotoxin treatment × postnatal day interaction ($F_{2,84}$ = 0.338, $p$ = 0.715; $n$ = 22 control mice; $n$ = 22 picrotoxin-exposed mice). Both treatment groups exhibited a progressive decrease in the latency to avoidance on P7, P9, and P11 ($F_{1.362,57.19}$ = 9.464, $p$ = 0.0013). No significant difference was found in the latency to fall between control and picrotoxin-exposed female mice on P25 ($t_{42}$ = 0.2545, $p$ = 0.8002; $n$ = 22 control mice; $n$ = 22 picrotoxin-exposed mice).

*Effects of prenatal exposure to picrotoxin on affective-like behaviors*

Picrotoxin-exposed female mice exhibited a decrease in active social interaction time compared with control female mice ($t_{42}$ = 0.111, $p$ = 0.912; $n$ = 22 control mice; $n$ = 22 picrotoxin-exposed mice). A trend toward a difference in the latency to flicking, jumping, and licking paws in the hot plate test was found between control and picrotoxin-exposed female

mice at 6-7 weeks of ages ($t_{42}$ = 1.795, $p$ = 0.080; $n$ = 22 control mice; $n$ = 22 picrotoxin-exposed mice). A significant difference in the number of grooming episodes in the self-grooming test was found between control and picrotoxin-exposed female mice ($t_{38}$ = 2.841, $p$ = 0.007; $n$ = 17 control mice; $n$ = 23 picrotoxin-exposed mice). No significant difference in grooming time in the self-grooming test was found between control and picrotoxin-exposed female mice ($t_{19.691}$ = 2.463, $p$ = 0.023; $n$ = 17 control mice; $n$ = 23 picrotoxin-exposed mice). No significant difference in the total distance travelled in the open field test was found between control and picrotoxin-exposed female mice ($t_{42}$ = 0.253, $p$ = 0.802; $n$ = 22 control mice; $n$ = 22 picrotoxin-exposed mice; Table S2). No significant difference in the time spent in the peripheral area was found between control and picrotoxin-exposed female mice ($t_{42}$ = 0.542, $p$ = 0.591; $n$ = 22 control mice; $n$ = 22 picrotoxin-exposed mice). A trend toward a difference in the number of turning episodes in the open field test was found between control and picrotoxin-exposed female mice ($t_{42}$ = 1.851, $p$ = 0.071; $n$ = 22 control mice; $n$ = 22 picrotoxin-exposed mice). No significant difference in the time spent on the open arms was found between control and picrotoxin-exposed female mice ($t_{42}$ = 0.907, $p$ = 0.369; $n$ = 22 control mice; $n$ = 22 picrotoxin-exposed mice). In adulthood, no significant difference in active interaction time in the social interaction test was found between control and picrotoxin-exposed female mice ($t_{41}$ = 0.134, $p$ = 0.894; $n$ = 22 control mice; $n$ = 21 picrotoxin-exposed mice). Body weight was recorded immediately before the social interaction test. No significant difference in body weight was found between control and picrotoxin-exposed female mice in adolescence (5-6 weeks of age; $t_{42}$ = 0.378, $p$ = 0.707). In adulthood, no significant difference in brain weight was found between control and picrotoxin-exposed female mice (10-11 weeks of age; $t_{41}$ = 0.389, $p$ = 0.699).

# Table S2. Results of behavioral tests in female mice.

| Mesurement items | Control mice | Picrotoxin-exposed mice | *p* |
|---|---|---|---|
| Body weight from P7-25 (g)* | P7, 3.8 (0.07), P25, 11.8 (0.17) | P7, 3.9 (0.07), P25, 11.3 (0.21) | Groups x Day, 0.010, Day, <0.0001, Groups, 0.197 |
| Eye-opening score | P12, 0, P13, 0.3, P14, 1.8, P15-18, 2 | P12, 0, P13, 0.6, P14, 1.9, P15-18, 2 | P12, >0.999, P13, 0.302, P14, 0.607, P15-18, >0.999 |
| Negativegeotaxis test (s) | P7, 5.9 (0.51), P9, 5.4 (0.55), P11, 4.9 (0.42) | P7, 6.4 (0.63), P9, 4.8 (0.50), P11, 5.4 (0.44) | Groups x Day, 0.489, Day, 0.077, Groups, 0.745 |
| Righting reflex test (s) | P7, 1.9 (0.24), P9, 0.9 (0.09), P11, 0.4 (0.04) | P7, 2.0 (0.37), P9, 0.9 (0.10), P11, 0.5 (0.04) | Groups x Day, 0.993, Day, <0.0001, Groups, 0.779 |
| Cliff avoidance test (s) | P7, 1.1 (0.10), P9, 0.8 (0.04), P11, 0.82 (0.05) | P7, 1.3 (0.17), P9, 0.8 (0.06), P11, 0.9 (0.07) | Groups x Day, 0.715, Day, 0.0013, Groups, 0.037 |
| Hanging wire test (s) | 119.1 (16.33) | 130.5 (20.46) | 0.662 |
| Social interaction test 5-6 wks of age (s) | 100.5 (5.47) | 102.0 (12.36) | 0.912 |
| Hot plate test (s) | 9.9 (2.13) | 8.7 (2.13) | 0.080 |
| Grooming test-total (number) | 25.0 (2.84) | 16.3 (1.58) | 0.007 |
| Grooming test-total (s) | 47.7 (8.22) | 26.3 (2.78) | 0.023 |
| Open field test, total distance (m) | 68.3 (2.14) | 69.2 (2.90) | 0.802 |
| Open field test, time spent in a marginal area (s) | 832.2 (21.72) | 815.4 (22.03) | 0.591 |
| Open field test, turning (number) | 9.5 (0.75) | 11.8 (0.93) | 0.071 |
| Elevated plus-maze test (s) | 65.8 (7.47) | 76.6 (9.30) | 0.369 |
| Social interaction test 10-11 wks of age (s) | 86.3 (5.67) | 84.95 (8.99) | 0.894 |
| Body weight in social interaction test in 5-6 wks and 10-11 wks of age (g) | 17.3 (0.90), 20.8 (0.28) | 17.2 (0.22), 20.7 (0.25) | 0.707, 0.699 |
| Brain weight (mg) | 464.0 (2.17) | 463.8 (3.37) | 0.969 |

*Body weight from P7-25 (g): The weight values on P7 and P25 are listed as representative values.

The figures in parentheses are SEMs.

*Sniffing ratio during active interaction*

We counted the time of sniffing, following, mounting, and grooming as active interactions. As shown in Table S3, the ratio of sniffing time during active interaction was approximately 90%.

Table S3. Sniffing ratio during active interaction at 10-11 weeks of age.

| | Control mice | Picrotoxin-exposed mice |
|---|---|---|
| **Sniffing/Active interaction (%)** | 95.4 (0.98) | 89.9 (3.21) |

The data in parentheses are SEMs.

**Supplementary Tables S4-S9**

These tables include the results of the pathway enrichment analysis and are listed in Excel files.

Table S4. Pathway enrichment of upregulated genes.

Table S5. Pathway enrichment of downregulated genes.

Table S6. Pathway enrichment of turquoise module.

Table S7. Pathway enrichment of blue module.

Table S8. Pathway enrichment of brown module.

Table S9. Pathway enrichment of gray module.

**Lack of side preference in turning behavior in the open-field test**

We counted the numbers of left and right turning episodes. As shown in Table S10, we did not find a side preference in either control or picrotoxin-exposed male mice ($t_{34}$ = 0.136, $p$ = 0.893; $n$ = 18 control mice, $t_{50}$ = 0.164, $p$ = 0.870; $n$ = 26 picrotoxin-exposed mice).

Table S10. Right and left turning behavior in control and picrotoxin-exposed male mice.

| | | **Control mice** | **Picrotoxin-exposed mice** |
|---|---|---|---|
| **Turning behaviors** | Right | 4.4 (0.58) | 5.7 (0.72) |
| | Left | 4.3 (0.28) | 5.6 (0.59) |

The data in parentheses are SEMs.
